# When Trimethoprim-Sulfamethoxazole Turns Pernicious: Type 4 Renal Tubular Acidosis in a Diabetic Patient With Sjögren’s Syndrome

**DOI:** 10.7759/cureus.91588

**Published:** 2025-09-04

**Authors:** Öykü Zeynep Gerçek, Birkan Alaycı, Fatih Borlu

**Affiliations:** 1 Internal Medicine, Şişli Hamidiye Etfal Training and Research Hospital, Istanbul, TUR; 2 Internal Medicine, Taksim Training and Research Hospital, Istanbul, TUR

**Keywords:** hyperkalemia, non-anion gap metabolic acidosis, pneumocystis jirovecii pneumonia, renal tubular acidosis, sjögren’s syndrome, trimethoprim-sulfamethoxazole

## Abstract

Type 4 renal tubular acidosis (RTA) is an underrecognized but potentially life-threatening complication characterized by hyperkalemic, non-anion gap metabolic acidosis. Trimethoprim-sulfamethoxazole (TMP-SMX), commonly used for treating *Pneumocystis jirovecii* pneumonia (PCP), has been implicated as a reversible trigger of this condition, especially in metabolically vulnerable patients. We report the case of a 63-year-old woman with type 2 diabetes mellitus and Sjögren’s syndrome who developed type 4 RTA during intravenous TMP-SMX therapy for PCP. Although initial arterial blood gas (ABG) analysis showed a near-normal pH, further evaluation revealed decreased serum bicarbonate (HCO₃⁻) and a partial pressure of carbon dioxide (PaCO₂) lower than expected by Winter’s formula, consistent with a mixed acid-base disorder. Hyperkalemia and a positive urine anion gap confirmed the diagnosis of type 4 RTA. Distal (type 1) RTA, commonly associated with Sjögren’s syndrome, was ruled out based on urine pH and potassium status. TMP-SMX was continued with close monitoring, and the acid-base abnormalities resolved following treatment completion. This case underscores the importance of considering medication-induced type 4 RTA in immunocompromised patients presenting with unexplained hyperkalemia and normal anion gap metabolic acidosis. TMP-SMX, although widely used and effective, necessitates vigilant electrolyte monitoring in susceptible individuals.

## Introduction

*Pneumocystis jirovecii *pneumonia (PCP), formerly known as *Pneumocystis carinii* pneumonia, is a life-threatening opportunistic infection classically seen in HIV-positive individuals with low CD4 counts. However, its incidence is increasingly reported in non-HIV populations, particularly among patients receiving immunosuppressive therapies. Corticosteroid use has long been recognized as a major risk factor. In a seminal study by Yale and Limper, a median prednisone dose of 16 mg/day over eight weeks was associated with a markedly increased PCP risk [1]. More recent data suggest that not only continuous steroid therapy but also intermittent high-dose regimens, chemotherapy, and underlying immunosuppressive or autoimmune conditions, even in the absence of treatment, can predispose patients to PCP [2,3]. Although the risk generally increases with cumulative dose and duration, lower daily doses over extended periods may still confer significant risk in vulnerable individuals.

Trimethoprim-sulfamethoxazole (TMP-SMX) remains the first-line therapy for PCP due to its proven efficacy. While gastrointestinal symptoms and cutaneous reactions are the most common adverse effects, TMP-SMX has also been associated with a range of renal complications [4]. Trimethoprim can cause a pseudo-rise in serum creatinine by inhibiting tubular secretion without true impairment in glomerular filtration rate. However, the sulfonamide component carries a risk of true acute kidney injury (AKI), either via hypersensitivity-related interstitial nephritis or, more rarely, through intratubular crystal deposition [5].

Beyond these well-known nephrotoxic effects, TMP-SMX has been implicated in the development of type 4 renal tubular acidosis (RTA), a form of hyperkalemic, non-anion gap metabolic acidosis resulting from impaired distal tubular ammonium excretion. Trimethoprim acts similarly to amiloride by inhibiting epithelial sodium channels (ENaC), thereby promoting potassium retention and mimicking a hypoaldosteronism-like state [6-8]. This disturbance can be particularly hazardous in patients with predisposing factors such as diabetes mellitus or chronic kidney disease. Although type 4 RTA remains underrecognized, it may lead to clinically significant hyperkalemia and acid-base disturbances, requiring prompt evaluation and intervention [9].

Here, we present the case of a woman with diabetes with Sjögren’s syndrome who developed type 4 RTA following TMP-SMX treatment for PCP. This case illustrates the importance of close electrolyte monitoring and highlights how coexisting autoimmune disease may contribute to tubular vulnerability. Clinicians should maintain a high index of suspicion for this complication, especially in immunocompromised individuals presenting with unexplained hyperkalemia and normal anion gap acidosis.

## Case presentation

A 63-year-old woman presented to the emergency department with a one-week history of progressively worsening fever, non-productive cough, shortness of breath, generalized malaise, diffuse myalgia, and new-onset confusion.

Her medical history included Sjögren’s syndrome, for which she had been receiving corticosteroids for nearly three weeks due to a recent flare, initially started at 10 mg/day and tapered down to 7.5 mg/day of prednisolone before the time of admission. She also had celiac disease, asthma, and a 10-year history of type 2 diabetes mellitus. Additionally, she had been hospitalized 10 years earlier for bacterial meningitis. Her regular medications included prednisolone, azathioprine, metformin, and albuterol as needed.

On examination, the patient was disoriented to time and place and appeared acutely ill. Her vital signs were notable for a temperature of 38.3°C, a blood pressure of 88/57 mmHg, a heart rate of 110 beats per minute, a respiratory rate of 30 breaths per minute, and an oxygen saturation of 89% while breathing room air. Respiratory examination revealed diffuse bronchial breath sounds and coarse crackles bilaterally.

Initial laboratory workup (Table 1) demonstrated a white blood cell count of 28,630/mm³ with 78% neutrophils, a hemoglobin level of 11.4 g/dL, a C-reactive protein of 334 mg/L, and a markedly elevated procalcitonin of 97.6 ng/mL. Serum electrolytes were within normal limits, with sodium at 134 mEq/L, potassium at 4.3 mEq/L, and glucose at 92 mg/dL. Renal function tests revealed a creatinine of 1.14 mg/dL and blood urea nitrogen of 18 mg/dL, while serum albumin was decreased to 2.8 g/dL. Liver enzymes were notable for alkaline phosphatase of 456 U/L, gamma-glutamyl transferase of 158 U/L, aspartate aminotransferase of 33 U/L, and alanine aminotransferase of 15 U/L. Arterial blood gas (ABG) analysis revealed a pH of 7.29, a partial pressure of carbon dioxide (pCO₂) of 55 mmHg, and a bicarbonate level of 25 mEq/L.

**Table 1:** Laboratory parameters at key clinical time points ABG: arterial blood gas, CRP: C-reactive protein, WBC: white blood cell count, AST: aspartate transaminase, ALT: alanine aminotransferase, ALP: alkaline phosphatase, GGT: gamma-glutamyl transferase, BUN: blood urea nitrogen, HCO₃⁻: bicarbonate, pCO₂: partial pressure of carbon dioxide, Na: sodium, K: potassium, Cl: chlorine, ER: emergency room, TMP-SMX: trimethoprim-sulfamethoxazole, IM: internal medicine

Parameters	Initial ER presentation	Day 6 of TMP-SMX (transfer to IM ward)	After discharge
pH	7.29	7.41	7.37
HCO3	25 mEq/L	18.6 mEq/L	23 mEq/L
pCO2 on ABG	55 mmHg	31.9 mmHg	-
CRP	334 mg/L	61.94 mg/L	10.2 mg/L
Procalcitonin	97.6 ng/mL	1.77 ng/mL	0.20 ng/mL
WBC	28,630/mm^3^	14,008/mm^3^	9,037/mm^3^
Albumin	2.8 g/dL	3.1 g/dL	3.5 g/dL
Na	134 mEq/L	134 mEq/L	137 mEq/L
K	4.3 mEq/L	6.4 mEq/L	4.5 mEq/L
Cl	99 mEq/L	111 mEq/L	100 mEq/L
AST	33 U/L	37 U/L	22 U/L
ALT	15 U/L	24 U/L	20 U/L
ALP	456 U/L	300 U/L	110 U/L
GGT	158 U/L	145 U/L	57 U/L
Creatinine	1.14 mg/dL	1.02 mg/dL	0.89 mg/dL
BUN	18 mg/dL	15 mg/dL	12 mg/dL

**Figure 1:**
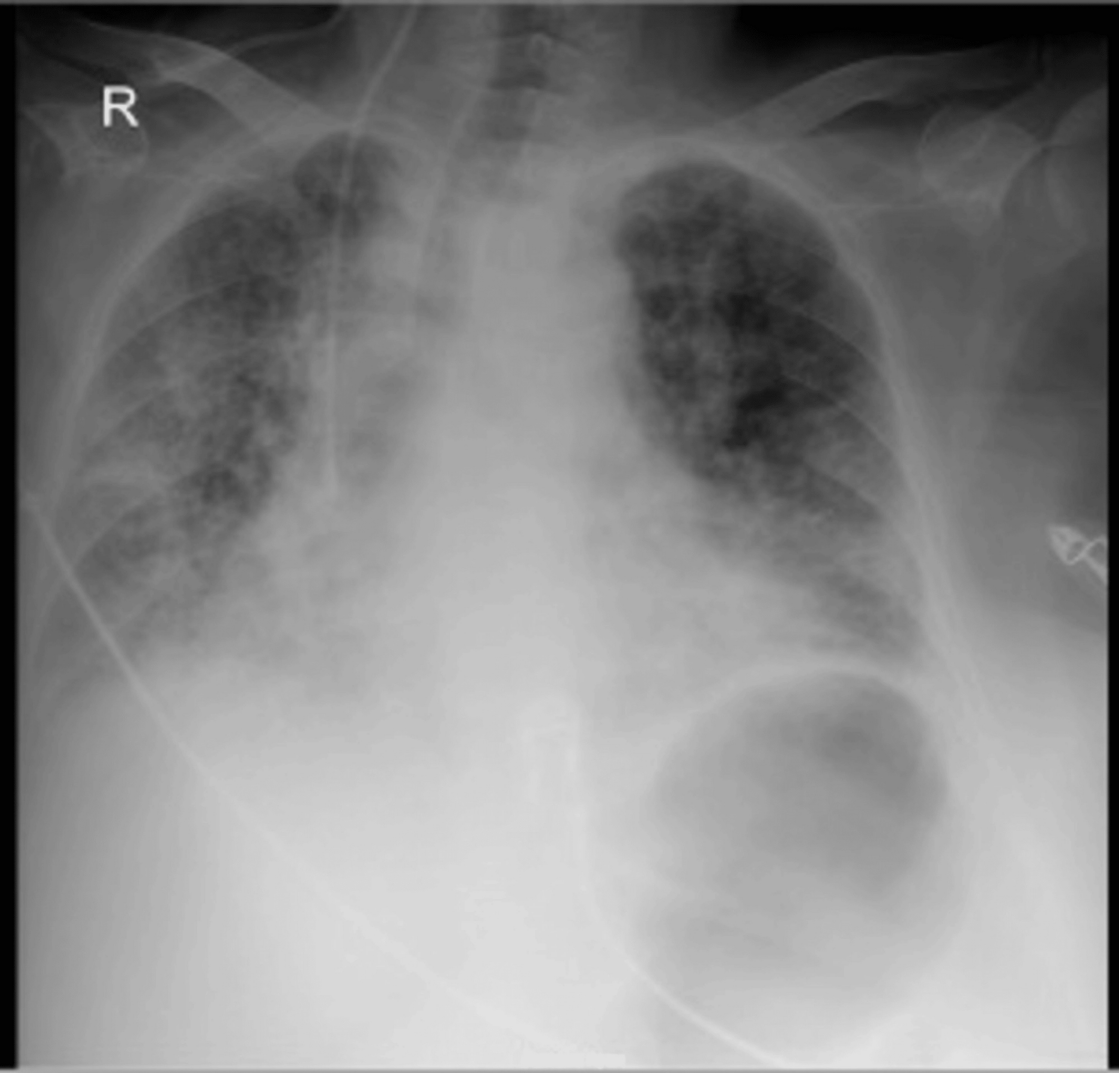
Initial chest X-ray demonstrating bilateral, diffuse micronodular perihilar infiltrates

Chest X-ray demonstrated bilateral, diffuse micronodular perihilar infiltrates (Figure 1).

The patient was diagnosed with sepsis and acute hypoxemic respiratory failure, intubated, and transferred to the intensive care unit. She was started on intravenous norepinephrine and empirically treated with teicoplanin 400 mg/day IV. In light of her ongoing corticosteroid therapy and clinical deterioration, IV dexamethasone was initiated to manage systemic inflammation related to sepsis while maintaining treatment for the recent autoimmune flare.

Two sets of blood cultures and an endotracheal aspirate culture were obtained, which remained negative.

Due to the patient’s immunocompromised status, non-resolving fever, lack of CRP decline by day 3, characteristic radiological pattern, and negative culture results, *Pneumocystis jirovecii* pneumonia (PCP) was suspected. An infectious disease consultation was obtained, and histochemical staining with methenamine silver revealed coccoid microorganisms in clusters, as well as morphologically characteristic *Pneumocystis jirovecii* organisms, supporting the diagnosis. Teicoplanin was discontinued, and the patient was started on intravenous trimethoprim-sulfamethoxazole (TMP-SMX) at 15 mg/kg/day TMP equivalent, divided into two daily doses. Dexamethasone was continued as adjunctive therapy for PCP, in line with standard treatment protocols.

An arterial blood gas analysis at this point, before the initiation of TMP-SMX, showed pH of 7.41, PaCO₂ of 42 mmHg, pO₂ of 90 mmHg, and HCO₃⁻ of 22 mEq/L.

**Figure 2:**
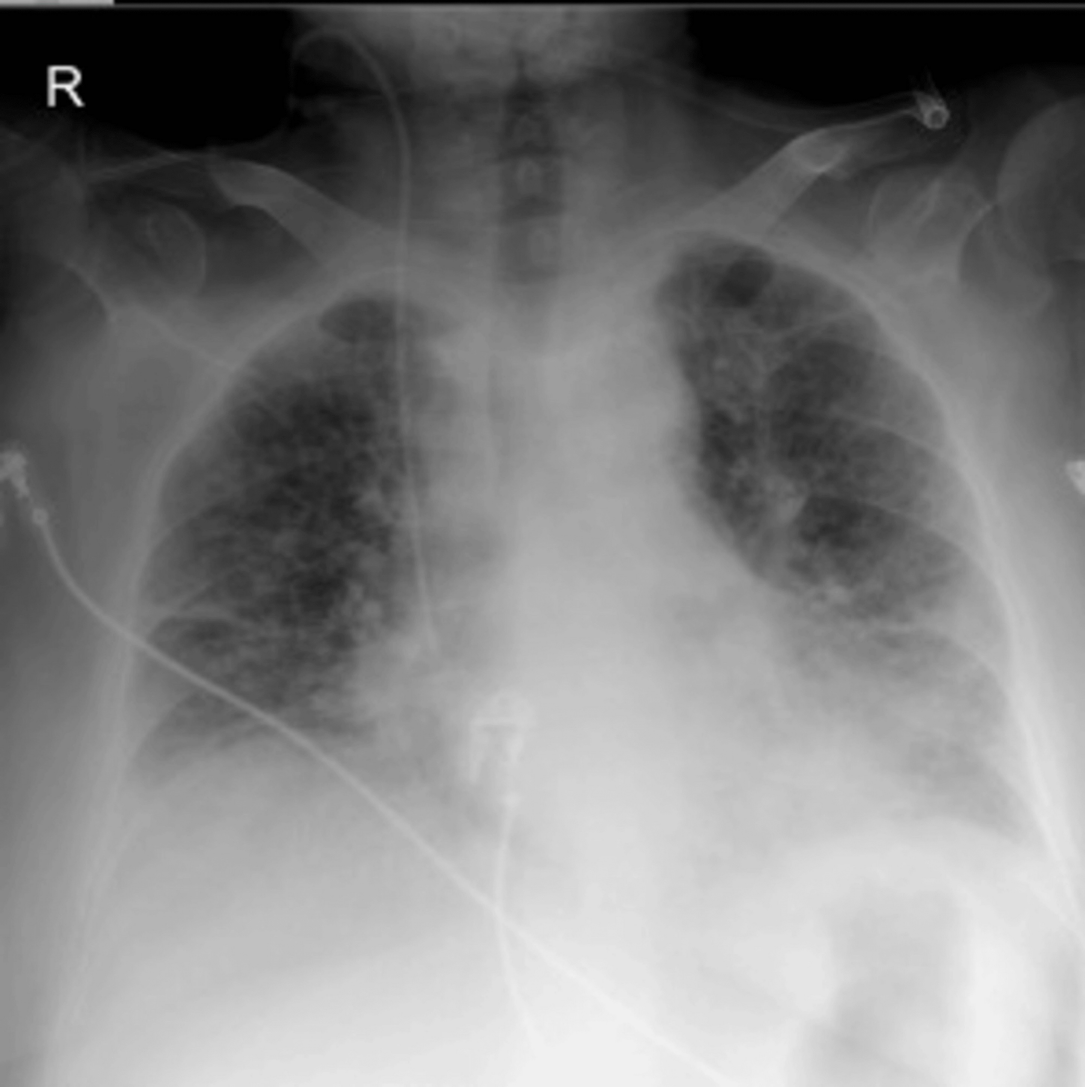
Follow-up chest radiograph obtained at this time demonstrating interval improvement in pulmonary infiltrates

By day 5 of targeted treatment, the patient showed significant clinical improvement, with resolution of fever and respiratory distress. She was successfully extubated and transferred to the internal medicine ward in stable condition. A follow-up chest radiograph obtained at this time demonstrated interval improvement in pulmonary infiltrates (Figure 2).

At the time of transfer to the ward, laboratory markers of inflammation had improved compared with admission values, consistent with the patient’s overall clinical stabilization (Table 1).

Although liver enzymes had peaked at admission, persistent elevation in cholestatic markers (ALP: 300 U/L, GGT: 145 U/L, bilirubin: 3.10 mg/dL) prompted an upper right quadrant ultrasound to evaluate for hepatobiliary pathology, which revealed no abnormalities. These findings were attributed to sepsis-related hepatic dysfunction. Ten days later, all liver function tests had returned to baseline. Also of note is that, within this ultrasound scan, the size and heterogeneity of the kidneys were noted to be normal by the radiologist. 

Of note, arterial blood gas analysis later revealed a normal pH (7.41) with decreased bicarbonate (HCO₃⁻: 18.6 mEq/L). Using Winter’s formula, the expected PaCO₂ was calculated as (1.5 x 18.6) + 8 = 35.9 mmHg. The measured PaCO₂ was 31.9 mmHg, indicating a concurrent respiratory alkalosis. The respiratory alkalosis was attributed to PCP-related tachypnea.

The albumin-to-creatinine ratio was obtained at 380 mg/g. It was planned to re-evaluate after discharge because it was potentially linked to the septic, febrile state of the patient.

The metabolic acidosis was further evaluated. The patient was hyperchloremic (Cl: 111 mEq/L), and the anion gap was calculated as 134 - (111 + 18.6) = 4.4, indicating a non-anion gap metabolic acidosis. Diarrhea was excluded clinically. Serum potassium was 6.4 mEq/L, without ECG abnormalities. The patient had normal urine output and was treated with nebulized albuterol and intravenous insulin dextrose infusion, as well as a low-potassium diet. The hyperkalemia supported a diagnosis of renal tubular acidosis.

To differentiate between diarrhea and renal tubular acidosis, urine studies were obtained. Urine pH was 5.2. Urinary potassium was 9.86 mEq/L, sodium 40 mEq/L, and chloride 30 mEq/L. The urine anion gap was calculated as 40 + 9.86 - 30 = 19.86, indicating impaired ammonium excretion. These findings were consistent with type 4 renal tubular acidosis.

TMP-SMX was continued for a total of 14 days. The patient was discharged in good clinical condition with normalization of serum potassium. Follow-up 10 days later showed resolution of acid-base disturbance with pH of 7.37 and HCO₃⁻ of 23 mEq/L, further suggesting the culprit to be the TMP-SMX treatment (Table 1).

## Discussion

Our case highlights a rarer renal side effect of TMP-SMX therapy, type 4 renal tubular acidosis (RTA), occurring in the setting of predisposing factors of diabetes and Sjögren’s syndrome.

Although our patient’s arterial blood gas (ABG) analysis initially revealed a normal pH of 7.41, closer inspection showed a decreased bicarbonate level (HCO₃⁻: 18.6 mEq/L), prompting further evaluation for an underlying metabolic acidosis.

The presence of a normal pH alongside reduced bicarbonate suggested the possibility of a mixed acid-base disorder. To assess appropriate respiratory compensation, Winter’s formula was applied: (1.5 × HCO₃⁻) + 8 = 35.9 mmHg. The patient’s measured PaCO₂ was 31.9 mmHg, lower than the expected value, confirming a concurrent primary respiratory alkalosis. This respiratory alkalosis was attributed to tachypnea in the setting of *Pneumocystis jirovecii* pneumonia-associated sepsis [10].

Further evaluation of the metabolic acidosis revealed a normal anion gap (Na⁺ - (Cl⁻ + HCO₃⁻) = 134 - (111 + 18.6) = 4.4), consistent with a non-anion gap metabolic acidosis. Diarrhea was excluded clinically, prompting consideration of RTA. The patient’s serum potassium level was elevated at 6.4 mEq/L, without electrocardiographic changes, which further supported this differential diagnosis.

Urine studies were obtained to distinguish between gastrointestinal bicarbonate loss and renal tubular dysfunction. Urine pH was 5.2, arguing against distal (type 1) RTA. The urine anion gap, calculated as (Na⁺ + K⁺ - Cl⁻) = 40 + 9.86 - 30 = 19.86 mEq/L, indicated impaired renal ammonium excretion. These findings, in the context of hyperkalemia and preserved urine output, were diagnostic of type 4 RTA.

After confirming the diagnosis, we examined the likely etiology. The patient had a long-standing history of diabetes, a known predisposing factor for type 4 RTA; however, previous clinical encounters had not revealed any acid-base disturbances. Renal function parameters, including proteinuria assessment, renal ultrasonography, and biochemical profiles, were not suggestive of diabetic nephropathy. The close temporal relationship between TMP-SMX initiation and the onset of electrolyte and acid-base derangements pointed toward the drug as the precipitating factor.

The diagnostic hallmarks of type 4 RTA are non-anion gap metabolic acidosis, hyperkalemia, and urine pH < 5.5. Common etiologies are listed in Table 2.

**Table 2:** Selected causes of type 4 renal tubular acidosis ACE: angiotensin-converting enzyme, ARBs: angiotensin II receptor blockers, NSAIDs: nonsteroidal anti-inflammatory drugs Source: Bonner R, Hladik G: Renal tubular acidosis: core curriculum 2025. Am J Kidney Dis. 2025, 85:501-12. 10.1053/j.ajkd.2024.08.014 [8]

Category	Example
Medications	β-adrenergic receptor blockers
	ACE inhibitors
	ARBs
	Mineralocorticoid receptor antagonists
	Direct renin inhibitors
	NSAIDs
	Calcineurin inhibitors
	Heparin and heparin analogs
Systemic diseases	Chronic kidney disease
	Diabetes mellitus
	Primary adrenal insufficiency
Genetic disorders	Congenital hypoaldosteronism (21-hydroxylase deficiency, isolated hypoaldosteronism)
	Pseudohypoaldosteronism type 2 (Gordon’s syndrome)

From a pathophysiological standpoint, type 4 RTA results from mineralocorticoid deficiency or resistance, leading to reduced stimulation of basolateral Na⁺/K⁺ ATPase and apical epithelial sodium channels (ENaC). This impairs proton excretion via apical H⁺-ATPase and promotes potassium retention. Chronic hyperkalemia further suppresses ammonium production in the proximal tubule, resulting in an acidic urine (pH < 5.5) despite impaired distal acidification [8].

Management in our case initially focused on correcting hyperkalemia, given its arrhythmogenic potential and its role in perpetuating the acidosis. TMP-SMX therapy was not discontinued because no better therapeutic alternatives for PCP were readily available [9].

Hypokalemic, distal subtypes of RTA are the dominant forms seen in the course of Sjögren’s syndrome. While Sjögren’s syndrome is classically associated with distal RTA, the clinical and biochemical profile in our patient pointed instead to type 4 RTA. This distinction was supported by the low urine pH (5.2), which is atypical for distal RTA, and the presence of hyperkalemia with a positive urine anion gap, hallmarks of impaired distal sodium-potassium exchange. In this case, type 4 RTA was likely multifactorial, with underlying diabetes as a predisposing factor and trimethoprim-sulfamethoxazole as the precipitating agent through ENaC blockade [8,9].

While type 4 RTA is a recognized complication of diabetes, several factors suggest that TMP-SMX was the primary trigger in our patient. The temporal association between drug initiation and onset of metabolic derangements, along with complete resolution after discontinuation, supports a medication-induced process. The preserved renal function and absence of known diabetic nephropathy make isolated hyporeninemic hypoaldosteronism less likely. Trimethoprim’s well-documented amiloride-like effect in the distal nephron, leading to impaired potassium and hydrogen excretion, aligns with the clinical course in this patient [10-12].

Reports of TMP-induced type 4 RTA remain rare in the literature. A similar case was described by Yavaşoğlu et al. in a patient receiving R-CHOP chemotherapy (rituximab, cyclophosphamide, hydroxydaunorubicin, Oncovin, and prednisone) for diffuse large B-cell lymphoma and TMP-SMX prophylaxis for PCP [13]. Another report described TMP-induced type 4 RTA in a patient with established diabetic nephropathy [14].

The key takeaway from this case is the need for vigilance in monitoring acid-base and electrolyte status, especially in patients on TMP-SMX who present with unexplained hyperkalemia and acidosis.

## Conclusions

Type 4 renal tubular acidosis is an underrecognized but potentially serious complication of diabetes, often overlooked due to an apparently preserved glomerular filtration rate. In our patient, a normal-range pH resulting from a mixed acid-base disorder with concomitant respiratory alkalosis masked the underlying metabolic acidosis and added to the diagnostic complexity. Although TMP-SMX has been a cornerstone in the treatment of several infections for more than five decades, its potential to cause life-threatening complications such as hyperkalemia warrants careful monitoring, particularly in high-risk populations.
